# Intraoperative mixed reality support for endoscopic submucosal dissection of a large laterally spreading tumor in the rectum

**DOI:** 10.1055/a-2639-4693

**Published:** 2025-08-20

**Authors:** Takeshi Uozumi, Maki Sugimoto, Yasuhiko Mizuguchi, Naoya Toyoshima, Seiichiro Abe, Ryuji Hamamoto, Yutaka Saito

**Affiliations:** 1 68380 Endoscopy Division, National Cancer Center Hospital, Tokyo, Japan; 2 Innovation Lab, Teikyo University Okinaga Research Institute, Tokyo, Japan; 3 Division of Science and Technology for Endoscopy, National Cancer Center Hospital, Tokyo, Japan; 4 13543 Division of Molecular Modification and Cancer Biology, National Cancer Center Research Institute, Tokyo, Japan; 5 Endoscopy Division, National Cancer Center Hospital, Tokyo, Japan

Mixed reality is a novel technology that adds digital elements to the real world to blend virtual and physical experiences. Recently, mixed reality has gained attention as a real-time intraoperative support modality that enhances the operator’s spatial awareness for detecting organs and vessels 1 . Colorectal endoscopic submucosal dissection (ESD) is widely used as a minimally invasive treatment; however, owing to its technical difficulty, endoscopists experience various levels of stress during the procedure regarding the possibility of perforation and bleeding. Mixed reality might help endoscopists accurately comprehend anatomical structures during ESD, preventing intraoperative adverse events without endoscopist stress. This is the first case report of intraoperative mixed reality support during colorectal ESD.

A 71-year-old woman was diagnosed with a 70-mm early colorectal cancerous tumor (cTis-T1a) in the lower rectum, and ESD was planned ( Fig. 1 ). Preoperative three-phase contrast-enhanced computed tomography (CT) images revealed three thick, penetrating vessels underneath the tumor. Before ESD, standard triangulated language formats of the rectum, vessels, and tumor were created from CT images and uploaded to the Holoeyes MD system (Holoeyes Inc., Tokyo, Japan) to create virtual three-dimensional (3D) models 2 .

**Fig. 1:**
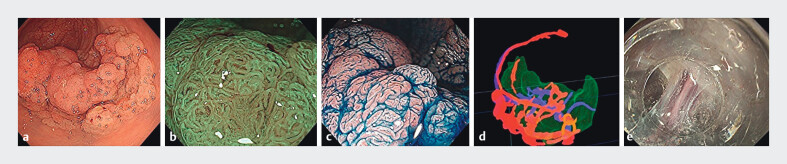
Preoperative and intraoperative images. **a** 70-mm 0-Is+IIa lesion (laterally spreading tumor, granular type: LST-G nodular mixed type) located in the lower rectum. **b** Japan Narrow Band Imaging Expert Team: JNET Type2A. **c** Peripheral intravenous therapy applied. **d** 3D model (red: artery; blue: vein; green: tumor). **e** Thick penetrating vessels are detected underneath the tumor during endoscopic submucosal dissection.

ESD was performed using a see-through head-mounted display (HoloLens; Microsoft Corporation, Redmond, Washington, USA) that enabled the superposition of a CT-based 3D model onto the real world ( Fig. 2 ). During ESD, as recognized in the CT-based 3D MR model, we identified the penetrating vessels and safely avoided major bleeding without stress ( Video 1 ). ESD was completed without any intraoperative adverse events, and the pathological diagnosis was intramucosal cancer with R0 resection.

**Fig. 2:**
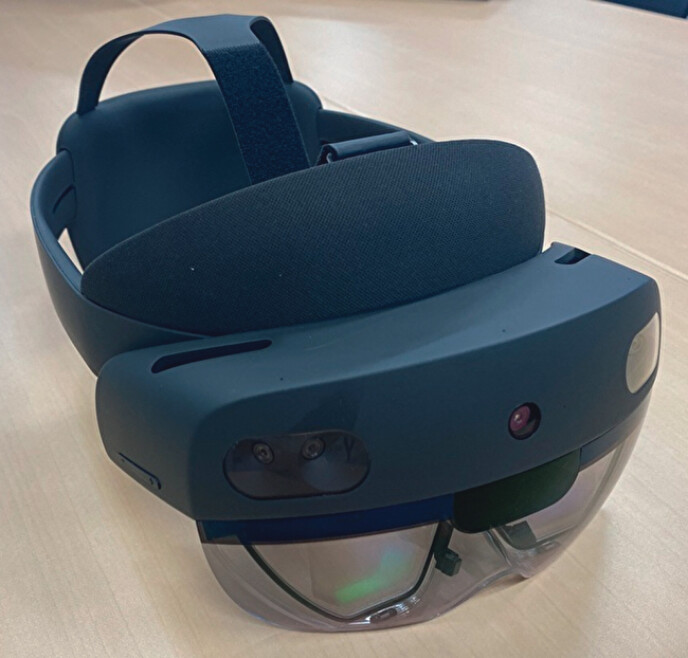
The see-through head-mounted display.

Intraoperative mixed reality support for endoscopic submucosal dissection.Video 1

In this case report, the intraoperative mixed reality support contributed to promptly comprehending the spatial relationships between the tumor and thick penetrating vessels, and we successfully avoided intraoperative bleeding without stress. The intraoperative mixed reality support might be a next-generation ESD support modality.

Endoscopy_UCTN_Code_TTT_1AQ_2AD_3AD

## Notes

Endoscopy E-Videos https://eref.thieme.de/e-videos
*E-Videos* is an open access online section of the journal *Endoscopy* , reporting on interesting cases and new techniques in gastroenterological endoscopy. All papers include a high-quality video and are published with a Creative Commons CC-BY license. Endoscopy E-Videos qualify for HINARI discounts and waivers and eligibility is automatically checked during the submission process. We grant 100% waivers to articles whose corresponding authors are based in Group A countries and 50% waivers to those who are based in Group B countries as classified by Research4Life (see: https://www.research4life.org/access/eligibility/ ). This section has its own submission website at https://mc.manuscriptcentral.com/e-videos .
